# Transcriptome profiling in the damselfly *Ischnura elegans* identifies genes with sex-biased expression

**DOI:** 10.1186/s12864-016-3334-6

**Published:** 2016-12-01

**Authors:** Pallavi Chauhan, Maren Wellenreuther, Bengt Hansson

**Affiliations:** 1 Department of Biology, Lund University, Lund, Sweden; 2 Institute for Plant and Food Research, Nelson, New Zealand

**Keywords:** *Ischnura elegans*, Odonata, Transcriptome, Sex differences, Gene expression, Olfaction, Pigmentation, Innate immunity

## Abstract

**Background:**

Sexual dimorphism occurs widely across the animal kingdom and has profound effects on evolutionary trajectories. Here, we investigate sex-specific gene expression in *Ischnura elegans* (Odonata: dragonflies and damselflies), a species with pronounced sexual differences including a female-limited colour polymorphism with two female-like gynochrome morphs and one male-mimicking, androchrome morph. Whole-organism transcriptome profiling and sex-biased gene expression analysis was conducted on adults of both sexes (pooling all females as well as separating the three morphs) to gain insights into genes and pathways potentially associated with sexual development and sexual conflict.

**Results:**

The *de novo* transcriptome assembly was of high quality and completeness (54 k transcripts; 99.6% CEGMA score; 55% annotated). We identified transcripts of several relevant pathways, including transcripts involved in sex determination, hormone biosynthesis, pigmentation and innate immune signalling. A total of 1,683 genes were differentially expressed (DE) between males and all females (1,173 were female-biased; 510 male-biased). The DE genes were associated with sex-specific physiological and reproductive processes, olfaction, pigmentation (ommochrome and melanin), hormone (ecdysone) biosynthesis and innate immunity signalling pathways. Comparisons between males and each female morph category showed that the gynochromes differed more from males than the androchrome morph.

**Conclusions:**

This is the first study to characterize sex-biased gene expression in odonates, one of the most ancient extant insect orders. Comparison between *I. elegans* sexes revealed expression differences in several genes related to sexual differences in behaviour and development as well as morphology. The differential expression of several olfactory genes suggests interesting sexual components in the detection of odours, pheromones and environmental volatiles. Up-regulation of pigmentation pathways in females indicates a prominent role of ommochrome pigments in the formation of the genetically controlled female colour polymorphism. Finally, the female-biased expression of several immunity genes suggests a stronger immune response in females, possibly related to the high levels of male mating harassment and recurrent matings in this species, both of which have been shown to injure females and expose them to sexually transmitted diseases and toxins contained in seminal fluids.

**Electronic supplementary material:**

The online version of this article (doi:10.1186/s12864-016-3334-6) contains supplementary material, which is available to authorized users.

## Background

Males and females of most species show pronounced phenotypic differences and divergent life-history traits that are under antagonistic selection pressures. Despite this, the majority of genes are shared between the sexes, which leads to strong sexual genetic correlations for many traits [1, 2]. Such genetic correlations can constrain the evolution of a trait towards a value that would be optimal for each sex, thus inhibiting the evolution of sexual dimorphism [3] and causing sexual conflict [1, 4–6]. This conflict may prevent the sexes from maximizing their fitness [7, 8]. The conflict may be resolved by the evolution of sexual dimorphism across different levels of ontogeny. In particular, sexual dimorphism can be mediated by sex-specific gene regulation, for example, through gene duplications, modifier genes, or dominance effects [9]. However, the evolution of sexual dimorphism may be constrained because many genes are likely to have pleiotropic effects and thus affect multiple traits simultaneously [10–13].

Central aspects of sex-specific gene regulation remain poorly understood in many animals, including in the ancient insect order Odonata (dragonflies and damselflies). We conduct novel gene expression analyses in adult male and female *Ischnura elegans* damselflies (family Coenagrionidae) to investigate sex differences in gene expression in general, and to identify transcripts of genes associated with pathways of potential importance for sexual development and sexual conflict in particular. *Ischnura elegans* is an evolutionary and ecological model species because of its ancient taxonomic position [14], the presence of a genetically controlled female-limited colour polymorphism [15, 16], and strong sexual conflict (reviewed in [17]). It is also an emerging odonate genetic model species, with the most complete male-specific annotated transcriptome of any odonate to date [18], a solid understanding of the inheritance of colour [16], a linkage map [19] and ongoing work to assemble the genome (Wellenreuther et al. in preparation). However, given the lack of a reference genome, it is not currently possible to investigate specific expression patterns and dosage compensation of genes located on the sex chromosome [20, 21].

**Fig. 1:**
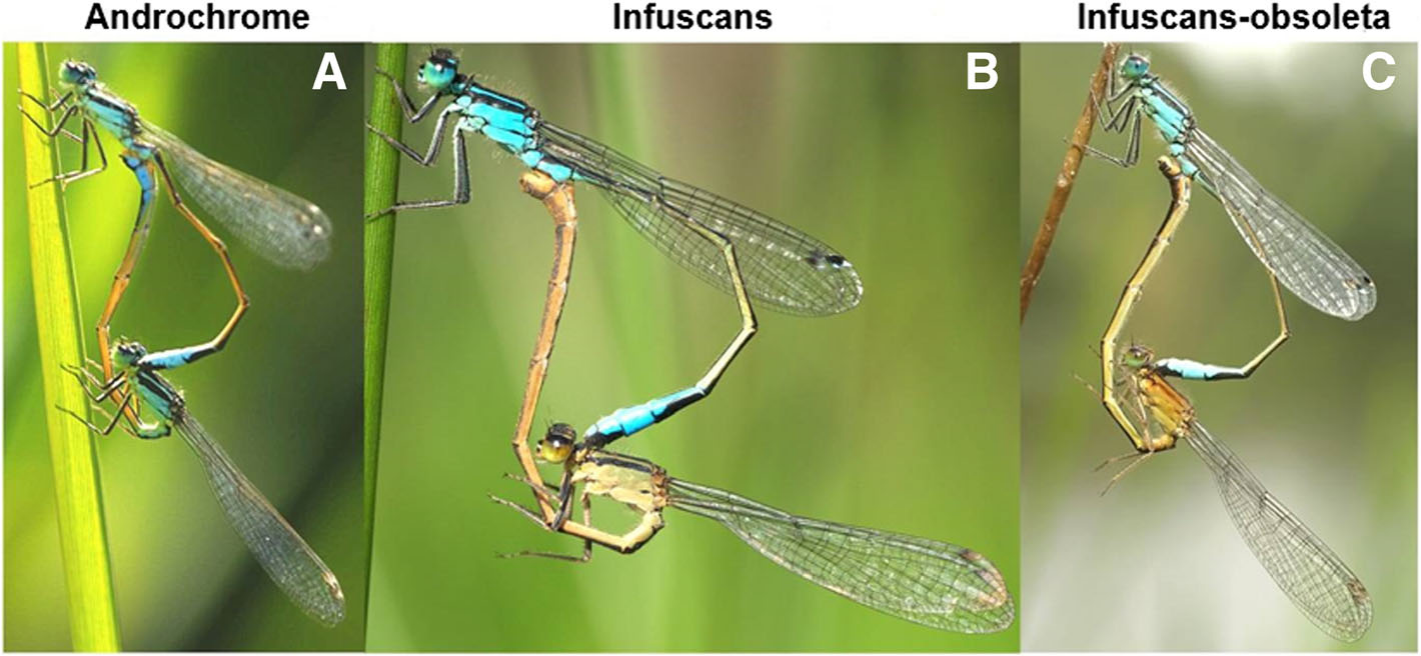
Mating wheels of *Ischnura elegans*. Males (always on the top of the mating wheel) with each of the species’ three female morph types; **a**) the androchrome, male mimicking morph, **b**) the infuscans morph and **c**) the infuscans-obsoleta morph

The colour polymorphism in *I. elegans* females [22, 23] is controlled by a Mendelian locus with three alleles in a dominance hierarchy [16]. Males are monomorphic in colour, whereas females occur in three different colour forms: the male-mimicking androchrome morph and the two more cryptic and female-like gynomorphs: infuscans and infuscans-obsoleta (Fig. 1). The colour polymorphism is widespread in the family Coenagrionidae, to which *I. elegans* belongs, and is described in more than 100 species [24, 25]. Several hypotheses have been proposed to explain the maintenance of the colour polymorphism, including negative frequency-dependent selection driven by male harassment [26–29]. The wide occurrence of the colour polymorphism among coenagrionid damselflies and its probable involvement in sexual conflict makes studying the differential gene expression in male and female damselflies a fascinating area of research [18, 30–32].

In this study, we identify transcripts of genes involved in key developmental and functional pathways by utilising information gathered from the assembled and annotated transcriptome. This allows us to gain insights into biological processes at the molecular level of both sexes. We also conduct differential gene expression analyses between males and females, and between males and each of the female morphs, to help understand the regulation of physiological, morphological and behavioural differences between the sexes and to derive knowledge about the genes and pathways that are potentially involved in sexual conflict.

## Results and discussion

### Assembly and annotation

#### Transcriptome sequencing

Sequencing of mRNA of 20 individuals generated a total of 215,374,125 paired-end 100 bp reads (44,643,077 reads from androchrome (*n* = 4), 41,044,225 reads from infuscans (*n* = 4), 57,779,728 reads from infuscans-obsoleta females (*n* = 5), and 71,907,095 reads from males (*n* = 7), respectively). After trimming of the adapters, low quality bases and removal of short reads, the total sequence set was reduced to 207,949,814 high quality reads (43,693,868, 39,718,852, 55,380,635 and 69,156,459 from the androchrome, infuscans, infuscans-obsoleta females, and males, respectively). A detailed summary of the raw reads and trimming step statistics can be found in the Additional file 1: Sheet 1. All 207,949,814 high quality reads obtained after trimming were used for the construction of the *de novo* transcriptome assembly.

#### De novo assembly and transcriptome assessment

The trimmed sequence reads from males and females were assembled into 127,858 transcripts using the Trinity software (version trinityrnaseq_r2014-07-17) [33]. The assembled contigs had a minimum length of 200 bp, N50 value of 1,989 bp and an average contig length of 850 bp. To improve the quality of the *de novo* assembly, a three-step quality filtering method was employed. In the first step, the transcripts were clustered at 95% sequence similarity to reduce sequence redundancy. This step clustered 5.2% transcripts together resulting in 121,151 unique transcripts. In the second step, poor quality and potentially mis-assembled transcripts were removed by excluding transcripts that showed a mean per base coverage of less than 5. After this step, a total of 54,375 high quality transcripts were retained. Finally, 68 transcripts containing ribosomal RNA were removed from the transcriptome with Repeatmasker [34]. The retained *de novo* transcriptome consisted of 54,307 high quality transcripts, with a contig N50 of 2,582 bp and an average contig length of 1,360 bp. A detailed description of the initial and final assembly statistics can be found in Additional file 1: Sheet 2. In the absence of a reference genome, the sequence completeness of the transcriptome assembly was assessed with CEGMA (Core Eukaryotic Gene Mapping Approach) [35]. Out of 248 ultra-conserved core proteins 247 were identified as “complete”, giving the transcriptome a completeness score of 99.6%. The remaining one was identified as “partial”. This indicates that the *de novo* assembly is almost complete, ensuring a high quality for all subsequent analyses.

#### Transcriptome annotation

To annotate the assembly, all high quality transcripts were blasted against the NCBI non-redundant (nr) protein database using BLASTX with an E-value cut-off of 1e-5 (i.e. 1*10^−5^). A total of 21,215 (39.1%) transcripts were reported to have at least one positive hit. Putative conserved protein domains in the high quality assembled transcripts were identified using BLAST2GO InterProScan, and 24,718 (45.5%) transcripts had at least one InterProScan annotation [36]. The list of the 20 most abundant InterPro domains is given in the Additional file 1: Sheet 3. The BLASTX and InterProScan annotated transcripts were subsequently annotated with Gene Ontology (GO) terms into three major GO categories: Cell Component, Molecular Function and Biological Processes. A total of 17,030 (31.4%) transcripts were associated with at least one GO term; concerning the second level ontology, 7,103 transcripts were assigned to a Cell Component category, 15,076 transcripts to a Molecular Function category and 10,984 transcripts to a Biological Process category. A total of 1,889 different enzymes were also identified using the KEGG (Kyto Encyclopaedia of Genes and Genomes) database, and these enzymes were present in 108 different metabolic pathways.

#### Quality of de novo assembly

Trimming of the initial dataset from 215,374,125 to 207,949,814 paired reads improved the overall dataset quality, resulting in a Q20 value of 100% and a significant increase in the Q30% (Additional file 1: Sheet 1). The initial *de novo* assembly consisted of 127,858 transcripts with an N50 value of 1,989 bp and an average contig length of 850 bp. The three-step quality filtering methods applied to enrich the transcriptome with high quality transcripts yielded 54,307 transcripts having an N50 of 2,582 bp and an average contig length of 1,360 bp. Comparing the initial assembly with the final assembly showed that not only the redundancy of the assembly was decreased, but also that poor quality and potentially mis-assembled short transcripts were removed from the transcriptome. The CEGMA assembly completeness score yielded 99.6%. Hence, the *de novo* assembly appears to be of high quality (in terms of single transcript quality) and quantity (in terms of completeness). In terms of functional capability, altogether 33,006 (55.4%) of the transcripts could be associated with at least one annotation term.

### Differential gene expression between the sexes

#### Number of differentially expressed transcripts and genes

Differential expression analysis was performed between males (*n* = 7) and all females (*n* = 13), as well as between males and each of the three female morphs (androchrome: *n* = 4; infuscans: *n* = 4; infuscans-obsoleta: *n* = 5) separately, using DESeq2 [37]. The level of sexual differential expression of the transcripts (log_2_ fold change), and their significance (p-values and multiple-test adjusted p-values), are listed in Additional file 1: Sheet 4–7, and visualised in Additional file 2: Figure S1.

**Fig. 2:**
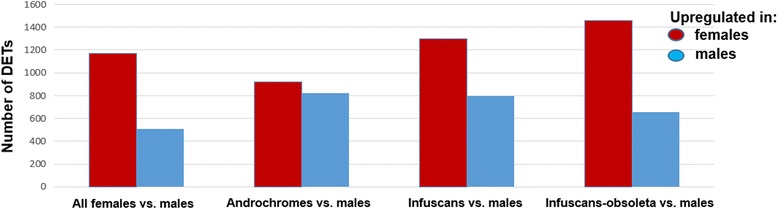
Sexual differential expression. Histograms showing the number of differentially expressed transcript (DETs) between males and all females, and between males and each of the female morphs. Red and blue bars refer to DETs that are up-regulated in either females or males, respectively. Data are given in Additional file 1: Sheet 8–11

For the analyses, we extracted the most differentially expressed transcripts (referred to as DETs hereafter) between males and females (listed in Additional file 1: Sheet 8–11; see Methods for details). This yielded 1,683 DETs that represented 1,296 differentially expressed genes (referred to as DEGs hereafter). Thus, some DETs were isoforms of the same gene. Among the 1,683 DETs, 1,173 were up-regulated in females and 510 in males (Fig. 2; Additional file 1: Sheet 8). The analyses between males and each of the three female morphs detected 1,740 DETs between androchrome females and males, 2,090 DETs between infuscans females and males, and 2,113 DETs between infuscans-obsoleta and males (Additional file 1: Sheet 9–11). Interestingly, a higher proportion of DETs were up-regulated in females than in males in the analyses of infuscans (62.0% up-regulated in females) and infuscans-obsoleta (68.9%) than in the analysis of androchromes (52.8%; Fig. 2). This result suggests that male-mimicking androchrome females are more similar to males in their gene expression than are the two gynochrome morphs.

We also performed Blast2GO enrichment analysis on selected DETs which resulted in six enriched GO terms between males and all females, five enriched GO terms between males and androchrome females, four GO terms between males and infuscans females, and one GO terms between males and infuscans-obsoleta females. The complete list of enriched GO terms is given in Additional file 1: Sheet 12. The KEGG biological pathways identified are reported in Additional file 1: Sheet 13.

#### Differentially expressed genes of specific interest

**Fig. 3:**
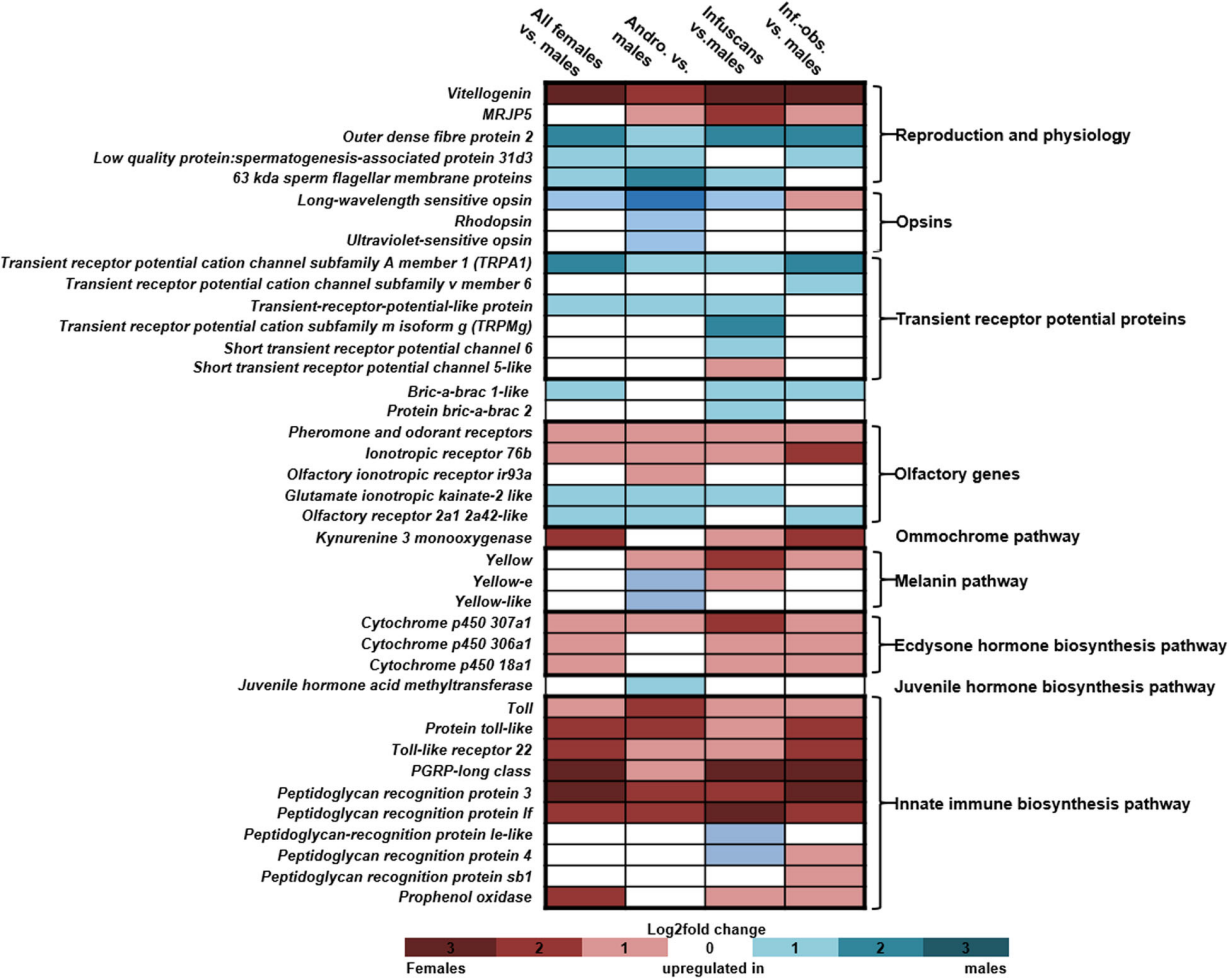
Heatmap of differentially expressed genes discussed in the manuscript (based on data in Additional file 1: Sheet 8–11). The log_2_ fold change-values from comparison between males and all females, and from males against each of the three female morphs, are indicated

Among the DEGs that were differentially expressed between males and females, we observed several genes that were associated with physiological and reproductive processes. For example, all analyses showed that the female morphs had a significant up-regulation of the *vitellogenin* gene (Fig. 3); a gene that produces vitellogenin protein, a precursor of lipoproteins and phosphoproteins involved in egg yolk production [38]. Studies on honey bees have demonstrated that *vitellogenin* not only plays an important role in oogenesis, but also regulates various behavioural (nursing and foraging) and survival traits (oxidative stress resilience, longevity and cell-based immunity) [39]. We also observed up-regulation of major royal jelly protein 5 (MRJP5) in all the female morphs (Fig. 3). In *Apis mellifera,* higher expression of MRJP5 in foragers than nurses suggested presence of some function in addition to regular brood food protein [40]. Among the genes that were up-regulated in males, we found several genes encoding for sperm proteins, including the outer dense fibre protein 2, low quality protein: spermatogenesis-associated protein 31d3, and 63 kda sperm flagellar membrane proteins (Fig. 3). These proteins are associated with the development of sperm tails, where they provide elasticity and protection from shear [41]. Interestingly, up-regulation of opsins was also observed in males with respect to androchrome and infuscans female morphs (Fig. 3). Higher expression of opsins in males suggests that males have better adaptability to distinguish colours than females, which in turn may be advantageous for mate selection, guarding territories from rival males, and food hunting [32]. In the assembled transcriptome, 24 transcripts encoded for different types of transient receptor potential proteins (TRPs; Additional file 1: Sheet 14). Among them, five different types of TRPs were up-regulated in males with respect to different female morphs (Fig. 3). While none of the TRPs were uniformly up-regulation in the female morphs. The generally higher expression of TRPs in males than females suggests a better capability of males to tolerate heat than females, as these receptors play an important role in thermal adaptations in insects [42, 43]. Moreover, we also found a male up-regulation of *bric-a-brac 1-like* (having BTB/POZ domain) against infuscans and infuscans-obsoleta female morphs. The closest homologue for the gene comes from *Tribolium castaneum*, where the associated protein is required for distal segmentation.

Because insects use olfaction for a variety of behaviours, including mate attraction, mate searching, oviposition site selection and the detection of host plant volatiles [44, 45], we looked specifically for olfactory genes in the assembled transcriptome. We identified several transcripts of odorant binding proteins (OBPs), chemosensory proteins (CSPs), odorant receptors (ORs) and ionotropic receptors (IRs) (Additional file 1: Sheet 15). Among them, genes encoding for one odorant and two ionotropic receptors were up-regulated in female morphs, while one ionotropic and one olfactory receptor were up-regulated in males (Fig. 3). Olfactory receptors play an important role in sex pheromone and odour detection, and their up-regulation in male suggests an essential role in sex pheromone detection in females [45]. Ionotropic receptors are ancient chemosensory receptors and respond to different odorants [46]. Differential expression of the various ionotropic receptors in the sexes suggests different behavioural response to environmental volatiles, e.g. Ionotropic receptor 76b helps in detection of low salts [47]. Overall, our finding of several ORs and IRs with a sex-biased expression in the sexes of *I. elegans* indicates that they play important sex-specific roles in pheromone and odour detection, and that studying their biological functions in vivo and in vitro would present an interesting venue for future studies.

### Differential gene expression within specific pathways

#### Differential gene expression in the ommochrome and melanin colour pathways

**Fig. 4:**
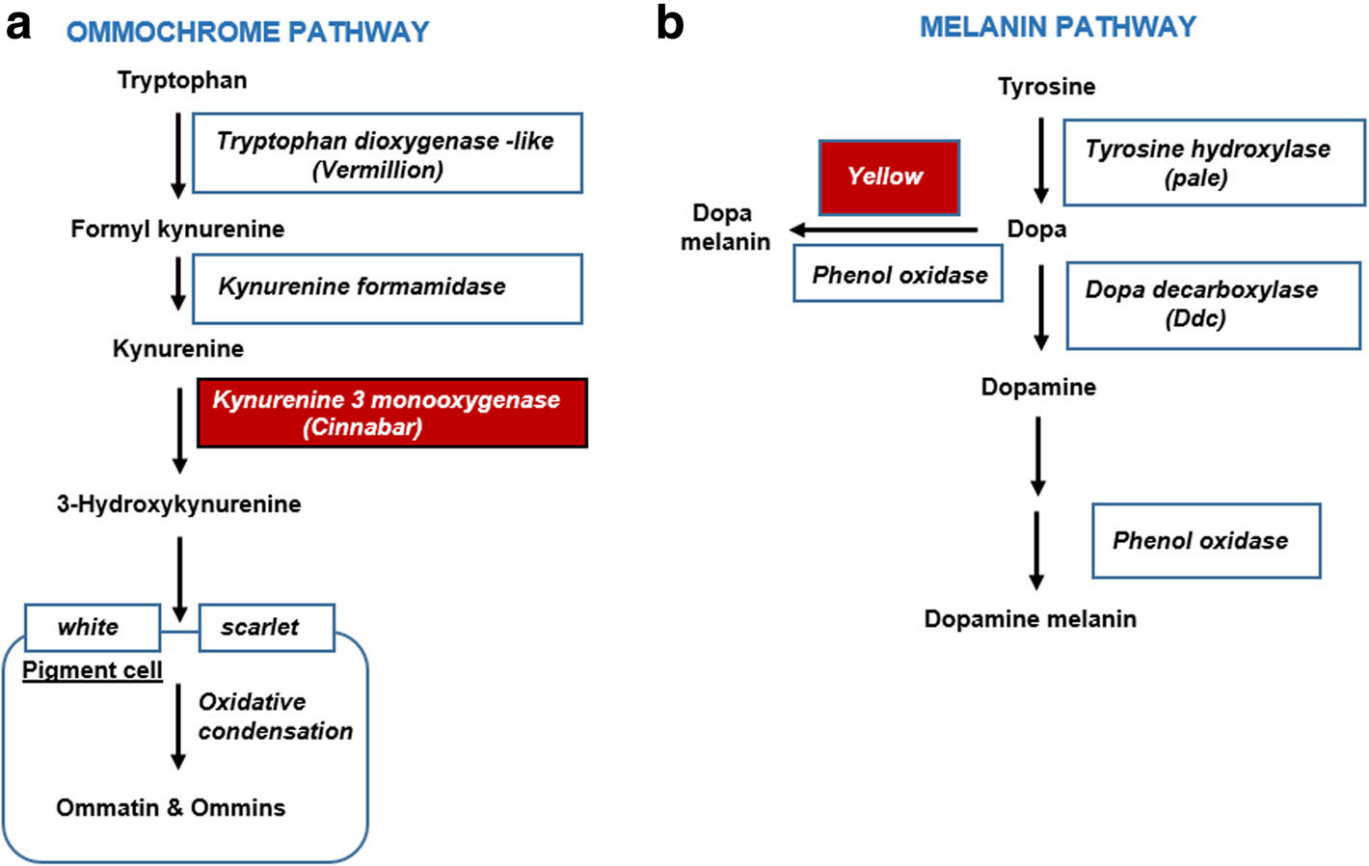
Ommochrome and melanin pigmentation pathway. Substrates/products (norm﻿a﻿l font) and genes/enzymes (*italics*) are given. Differentially expressed genes/enzymes are represented with red colour boxes. **a** In the ommochrome pigmentation pathway, kynurenine 3 monooxygenase was up-regulated in the two gynochrome morphs (i.e. the infuscans and infuscans-obsoleta morphs). **b** In the melanin pathway, the *yellow gene* was uniformly up-regulated in all the female morphs (see Fig. 3 and Additional file 1: Sheet 8–11)

We have previously identified the melanin, pteridine and ommochrome colour pathways in *I. elegans* [18]. To further understand the mechanisms that lead to colour differences in the sexes, we examined the occurrence of sex-biased genes in these pathways. No DEGs were found in the pteridine pathway, while the ommochrome and melanin pathway showed evidence for differential expression between the sexes. In the ommochrome pathway, the *cinnabar* gene, coding for the enzyme kynurenine 3-monooxygenase, was up-regulated in the two gynochrome morphs (i.e. the infuscans and infuscans-obsoleta morphs) (Fig. 3). The enzyme kynurenine 3-monooxygenase controls the conversion of kynurenine into 3-hydroxykynurenine, which then enters into the pigment granules and undergoes oxidative condensation and forms ommins and ommatins (ommochrome pigments) (Fig. 4a), suggesting a higher expression of kynurenine 3-monooxygenase and subsequently a higher accumulation of ommochrome pigments. Higher accumulation of these ommochrome pigments likely contributes to the formation of various light and dark colour pigments in gynochromes females, which may in turn contribute to the colour differences seen among the trimorphic females and monomorphic males. Ommatins have a low molecular weight and are alkaline labile compounds responsible for light colouration. Ommins, however, have a high molecular weight and are alkaline stable compounds. Mixtures of ommatins and ommins thus provide dark colours and shades in insects [48]. Our findings corroborate other studies on insect showing an importance of ommochrome pigments in insect colouration and polymorphism. For example, the pink colour of *Schistocerca* grasshoppers is due to a mixture of different ommochromes [49]. Similarly, in odonates, red colour is caused by the presence of red and brown colour ommochromes, while blue colour is produced by a dark brown ommochrome background. Finally, ontogenetic conversion of red dragonflies into yellow is caused by a redox-dependant colour change of ommochrome pigments [31, 50].

We observed differential up-regulation of *yellow* genes (melanin pathway) between the sexes (Fig. 4b). *Yellow* protein was differentially up-regulated in all the female morphs, while *protein yellow-like* and *yellow-e* showed up-regulation in the males and the androchrome female morph (Fig. 3). Studies on *Drosophila* spp. have suggested that changes in the expression of *yellow* gene correlate with different melanin patterns in the wings, abdomen and thorax [51, 52]. *Yellow-e* expression has been linked with pigmentation of *Bombyx* spp. larvae head and tail spots [53]. In future studies it would be interesting to examine experimentally if *yellow genes* indeed contribute to the colour differences between the sexes in *I. elegans*.

In the present study, we also found expression of genes coding for lycopene cyclase phytoene synthase (carRA) and phytoene desaturase/phytoene dehydrogenase (carB). This finding made it possible to identify the fourth colour pathway in *I. elegans*, the carotenoid pathway, which was not detected in our previous transcriptome study [18]. The biosynthesis pathway and annotation details are given in Additional file 2: Figure S2 and Additional file 1: Sheet 16. Carotene and xanthophyll, together constituting carotenoid pigments, are lipid soluble, containing no nitrogen and absorb light with green and blue wavelengths, which is producing a green, blue-green, blue and red colour to the insect integument and haemolymph [54]. Carotenoids play an important role in antioxidation, photoreception and colouration [50, 54]. However, none of the genes in this pathway were uniformly differentially expressed between the sexes.

#### Sexual differential expression of the hormone biosynthesis pathway

**Fig. 5:**
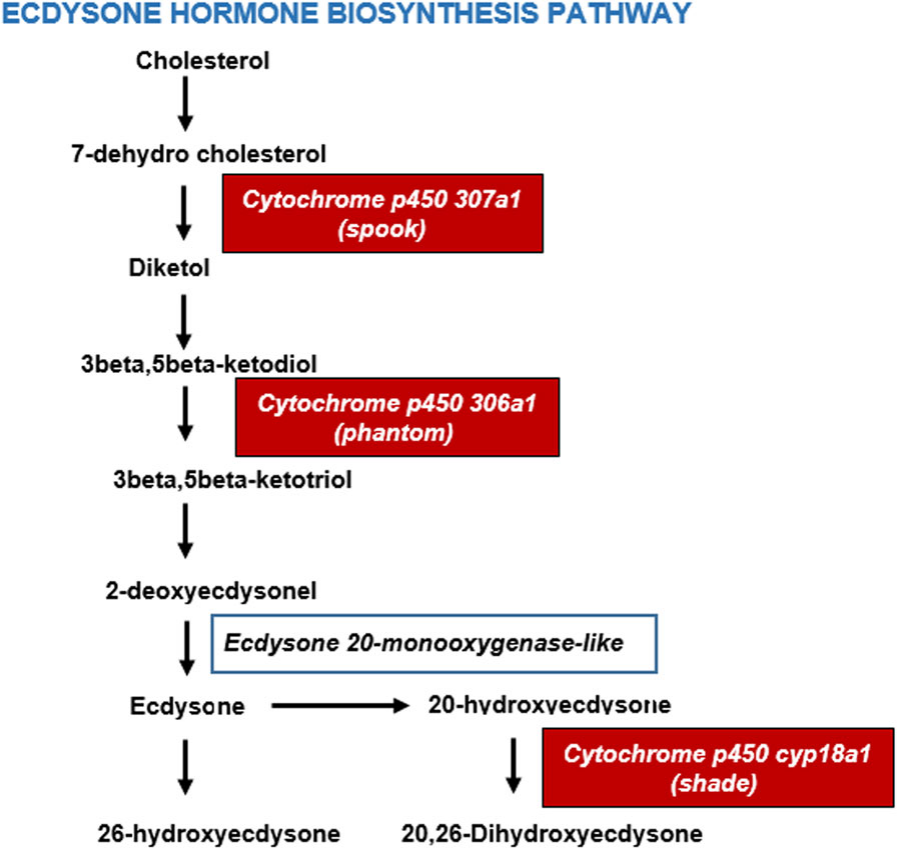
Ecdysone hormone biosynthesis pathway. Substrates/products (normal fon﻿t)﻿ and genes/enzymes (*italics*) are given. Differentially expressed genes/enzymes are represented with red colour boxes. Cytochrome p450 307a1 was uniformly up-regulated in all females (pooled) and in all female morphs, while Cytochrome p450 306a1 and Cytochrome p450 cyp18a1 were up-regulated in the two gynochrome morphs (see Fig. 3 and refer Additional file 1: Sheet 8–11)

Insect hormones have long been studied due to their importance in physiology, development and behaviour [55, 56]. Several studies on insect hormones have pinpointed their role in (i) the regulation of muscle activity such as in myotropic peptides, (ii) the regulation of reproduction, growth, and development, (iii) the pheromone biosynthesis (iv), diapause hormones, and (v) the stimulation or inhibition of tanning and of colour change (reviewed in [57]). In the present study, we were able to identify two hormone biosynthesis pathways, namely the juvenile hormone biosynthesis pathway and the ecdysone hormone biosynthesis pathway. Detailed annotations of genes and enzymes associated with these pathways are described in Additional file 1: Sheet 17. However, the genes in the juvenile hormone biosynthesis pathway were not uniformly differentially expressed between the sexes (but may be so earlier in ontogeny) (Additional file 2: Figure S3). Genes in the ecdysone biosynthesis pathway (Fig. 5) were, however, differentially expressed between sexes: cytochrome p450 307a1, cytochrome p450 306a1 and cytochrome p450 18a1 were up-regulated in females. The morph specific analyses between males and females showed that cytochrome p450 307a1 was up-regulated in all female morphs, while Cytochrome p450 306a1 and Cytochrome p450 cyp18a1 were up-regulated in the two gynochrome morphs (Fig. 3). High expression of these enzymes leads to higher production of edysone and 20-edysone in adult females, suggesting their requirement in proper development of follicles and oogenesis [58].

#### Females up-regulate the innate immune signalling pathway

**Fig. 6:**
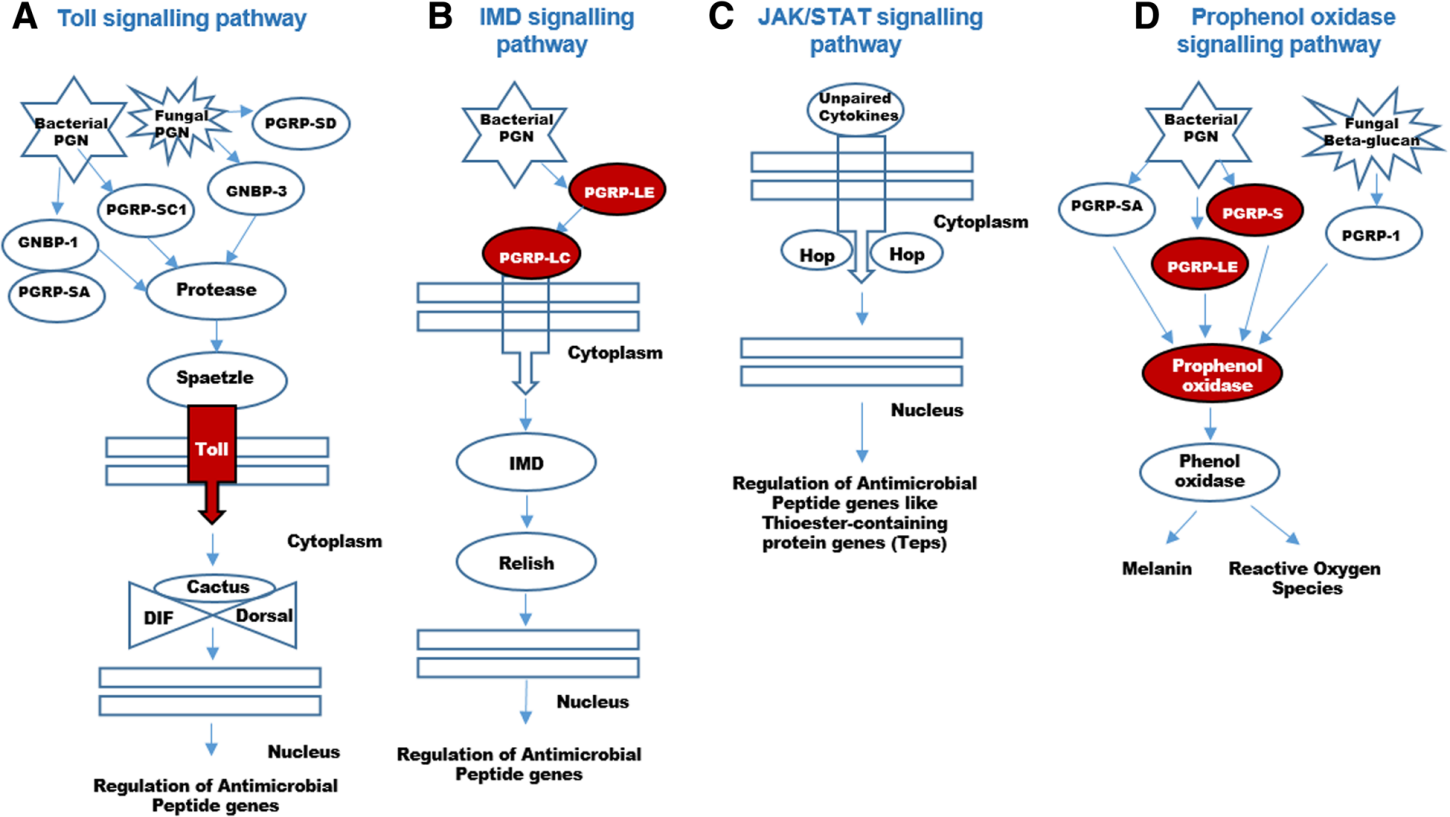
The four different types of innate immune signalling pathways recognised in *I. elegans*. Differentially expressed genes/proteins are represented with red colour boxes. **a** The Toll immune pathway: exposure to bacterial and fungal peptidoglycans trigger the production of insect peptidoglycan recognition proteins (PGRPs), like PGRP-SA, PGRP-SC1 and PGRP-SD, which in co-operation with Gram-negative binding proteins (GNBPs) like GNBP-1 and GNBP-3, activates proteases Spaetzle (a cytokine-like protein); which in turn activates Toll. Activated Toll activates Dorsal and Dif transcription factors, which then enter into the nucleus, bind to NfkB sites and initiate the transcription of antimicrobial peptides that kill Gram-positive bacteria and fungi by degrading their cell walls. **b** In the Imd pathway, exposure to meso-diaminopimelic acid (DAP) type peptidoglycan produced by Gram-negative bacteria activates PGRP-LE and PGRP-LC, which in turn activates Imd. Activated Imd further activates Fas-associated death domain (FADD) and death related ced-3/nedd2-like caspase (DREDD), which then activates Relish transcription factor. This enters into the nucleus and induce transcription of antimicrobial peptides detrimental for Gram-negative bacteria. **c** JAK/STAT cytokine receptor activation caused by unpaired ligands released during infection and injury causes phosphorylation of JAK tyrosine kinase Hopscotch (Hop). The phosphorylated Hop phosphorylate stat 92e, which then dissociate from receptor, dimerize, enters into the nucleus and induces transcription of Thiosester-containing protein genes (Teps) and Turandot (Tot) stress genes. **d** Prophenol-oxidase cascades are triggered upon exposure to bacterial (Lys and DAP peptidoglycan) and fungal (1, 3-beta-D-glucan) cell wall components. Activation of cascade produces PGPRs (PGRP-S, PGRP-LE and PGRP-1), which generate antimicrobial products like melanin and reactive oxygen species for defence. Refer Fig. 3 and Additional file 1: Sheet 8–11 for differential up-regulation components of immune system

The innate immune system acts as a first line of defence and protects hosts from a variety of foreign pathogens [59]. The four different types of innate immune signalling pathways recognised in insects are the (i) Toll, (ii) Immune deficiency (Imd), (iii) Janus kinase and the Signal Transducer and Activator of Transcription (JAK/STAT), and (iv) prophenol-oxidase cascade. The Toll immune pathway responds against Gram-positive bacterial and fungal infections, while the Imd pathway targets Gram-negative bacteria, and the JAK/STAT pathway becomes activated in response to infection and injury by bacteria and viruses [60]. Upon exposure to a specific infection, the corresponding insect peptidoglycan recognition proteins (PGRPs) are then activated, further triggering the signalling cascade [61]. Apart from these three pathways, PGPRs (PGRP-S, PGRP-LE and PGRP-1) also activate the prophenol-oxidase cascade. In the assembled transcriptome, we identified several genes of the four innate signalling pathways (Fig. 6). In addition, we identified a total of 27 transcripts encoding different PGRPs (Additional file 1: Sheet 18).

Peptidoglycan recognition protein 3 and peptidoglycan recognition protein long class (PGRP-LC) were among the most differentially up-regulated transcripts in all the female morphs. These proteins are usually activated in response to infection, but here their uniform up-regulation in all female samples indicates a general (rather than e.g. an infection related) up-regulation (see Additional file 1: Sheet 8). Apart from PGRP, we also observed female up-regulation of several other components of the innate immunity pathway, including prophenol oxidase activation factor, Toll, protein toll-like and toll-like receptor 22. The up-regulation of Toll in the Toll signalling pathway (Fig. 6a), PGRP-LC in the Imd signalling pathway (Fig. 6b) and prophenol oxidase (Fig. 6d) in the prophenol oxidase signalling pathway suggest the presence of a stronger innate immune system response in females compared to males (Fig. 3).

Studies on the polymorphic damselfly species *Coenagrion puella* have demonstrated that both sexes, as well as all three female morphs, are under equal pressure of parasitic infection [62]; yet our results suggest that *I. elegans* females have activated their immune defence to a higher extent than males. In many other invertebrate species it is also observed that females invest more in immunity than males [63, 64]. The difference in sex-specific immunity has been linked to Bateman’s principle which states that females can gain greater fitness by investing into immunity defence systems to maximize their lifespan, as increased immunity increases longevity and consequently the time for egg production and oviposition [65], whereas males gain greatest fitness by maximising their mating frequency with females. Hence, our results suggest that female morphs of *I. elegans* may have a higher baseline immunity level than males possibly to maintain a longer reproductive life. Moreover, it is also plausible that female *I. elegans* invest more in immune defence (compared to males) as a consequence of the excessive male mating harassment that is common in this species [27, 28, 66], and has been shown to cause injuries to females and expose females to toxins (present in the seminal fluids) and sexually transmitted diseases [67, 68].

#### The sex determination pathway: genes are expressed but not sex-biased

Sex determination is an important biological phenomenon that initiates a cascade of effects that leads to sex dimorphism in morphology, physiology and behaviour [69]. In insects, sex determination has been extensively studied in *Drosophila* spp. [70]. Considering *Drosophila* as a model organism, we were able to trace the sex determination pathway in *I. elegans* and identify all its components: *runt*, *daughterless*, *deadpan*, *sex lethal (Sxl)*, *transformer-2 (tra)*, *doublesex* (*dsx)*, *fruitless (fru)*, *fem* and *male-specific lethal 1, 2* and *3* genes (Additional file 2: Figure S4; detailed annotation information is given in Additional file 1: Sheet 19). However, with the exception of *doublesex* (up-regulated in males only in the comparison with androchrome females) none of these genes were differentially expressed between sexes. This was not surprising, since sex determination can often take place early during development and we have analysed exclusively adult *I. elegans*.

## Conclusions

This is the first study to characterize differential expression of genes in males and females of *I. elegans*. We successfully identified functionally important genes, pathways and processes involved in reproduction, olfaction, colouration, hormone and immune response and sex determination. These findings allow to gain insights into the phenotypic and behavioural differences between males and females. A main finding was the detection of sex-biased expression of several olfactory genes, which suggests that a sex-specific detection of pheromones, odours and behavioural responses to environmental volatiles is present. Furthermore, an interesting finding was a female-bias in several important functional pathways. The ommochrome pigmentation pathway was up-regulated in gynomorphs females, indicating a prominence of ommochrome pigments (ommins and ommatins) in the development of colour polymorphic females compared to the monomorphic males and the androchrome female morph. In addition, the hormone ecdysone biosynthesis pathway was female-biased, a finding that proposes a role of this pathway in the development of follicles and oogenesis. Finally, the up-regulation of immunity genes in females suggests a stronger immune response in females than in males, potentially to protect them from a wide range of pathogens (e.g. diseases and toxins) and also to buffer them against injuries resulting from multiple matings. In this species, females are exposed to excessive male mating harassment that causes injuries to them and exposes them to toxins and sexually transmitted diseases. This study provides a foundation for future genomic and transcriptomic studies in *I. elegans* as well as in other odonate and invertebrate species.

## Methods

### Species, collection and sample preparation

Seven males and 13 females (4 androchrome, 4 infuscans and 5 infuscans-obsoleta female colour morphs) of *I. elegans* were collected on the 20th of June 2011 in southern Sweden (Flackarp, 55°40′59, 13°09′52). Upon capture, all individuals were immediately killed by decapitation with a razor blade and subsequently stored in individual Eppendorf tubes in RNAlater (Qiagen). RNA was extracted with the Qiagen RNA extraction kit following the manufacturer’s specification. RNA from each individual was then separately subjected to paired-end sequencing with an Illumina HiSeq 2000 sequencer at the Beijing Genomics Institute Shenzhen in China.

### Raw data quality control and *de novo* assembly construction

Adapter and low quality bases having an average quality score of less than 20 were trimmed using the Nesoni clip version 0.109 [71]. After trimming, reads that had a read length of less than 25 bp were also discarded from the dataset. All high quality reads that remained after these steps were subsequently used for a *de novo* assembly. The Trinity version trinityrnaseq_r2014-07-17 was used for construction of the *de novo* assembly, using default parameters [33].

### Assembly quality filtering

The initial assembly generated by Trinity was subjected to a three-step quality filtering check to retain only high quality transcripts from the assembly. In the first step, duplicates were removed by clustering the assembly at 95% sequence similarity using CD-HIT-EST version 2.17.0 [72]. In the second step, poor quality and potentially mis-assembled transcripts were discarded from the assembly. In this step, the BED tools [73] genome coverage application was used to calculate the read coverage at each base, and then transcripts having a mean coverage per base less than five were removed from the assembly. In the third and final step, the transcripts containing ribosomal RNA were removed from the assembly. For this, the RepeatMasker version 4.0.1 was used in default mode to identify RNA and repetitive elements in the assembly [34]. The high quality assembly obtained after the three-step filtering was used for all subsequent analysis.

### Assembly annotation

To functionally annotate the assembly a BLASTX sequence homology search was conducted against the NCBI non-redundant (nr) protein database using an E-value cut-off of 1e-5. All the BLASTX results were then imported into BLAST2GO web start version for further annotations. BLAST2GO InterProScan was used for identifying conserved protein domains in the assembly. Then GO annotation was performed on the BLASTX and InterproScan annotated transcripts. The GO annotations were further refined into Biological Processes, Cellular Components and Molecular Functional annotations using second level database. GO_Slim reductions were also performed on GO terms to obtain more precise GO definitions. Further, enzymes and their corresponding biological pathways were identified in the assembly using the BLAST2GO integrated KEGG database. All the analyses were performed using default parameters. Blast2GO enrichment analysis (using Fisher’s Exact Test) was performed on DETs using default settings (False Discovery Rate; FDR < 0.05).

### Differential expression analysis

Differential expression analysis was performed using the Trinity pipeline “Identification and analysis of differentially expressed trinity genes and transcripts”. According to the pipeline, first the abundance estimation of the transcripts was performed using “Trinity: abundance estimation using RSEM or express and visualization using IGV” program with RSEM, one by one on all the four sets of filtered reads from female androchrome (*n* = 4), infuscans (*n* = 4), infuscans-obsoleta (*n* = 5) and males (*n* = 7). Then, identification of differentially expressed transcripts between males (*n* = 7) and females (*n* = 13), and males and female morphs (androchrome: *n* = 4; infuscans: *n* = 4; infuscans-obsoleta: *n* = 5), was performed using the Bioconductor tool DESeq2 [37]. Further, the most differentially expressed transcripts were extracted using the default cut-offs of the Trinity pipeline wherever applicable for the p-value (multiple adjusted *p* < 10^−3^) while the cut-off for log_2_ fold change was set to ≥1. All the analyses were performed using default parameters, with samples as replicates (i.e. samples were not pooled within categories).

All analyses were performed with the use of resources provided by SNIC through the Uppsala Multidisciplinary Center for Advanced Computational Science (UPPNEX) under Project number b2013227.

## Additional files

Additional file 1: Sheet 1. RNA sequence data trimming statistic. Sheet 2: Summary of initial and final *de novo* assembly. Sheet 3: 20 most abundant protein domain. Sheet 4–7: List of all transcripts with results from the differential expression analysis between all females and males (sheet 4), androchrome females and males (sheet 5), infuscans females and males (sheet 6), and infuscans-obsoleta females and males (sheet 7). Sheet 8–11: List of the most differentially expressed transcripts (DETs) between all females and males (sheet 8), androchrome females and males (sheet 9), infuscans females and males (sheet 10), and infuscans-obsoleta females and males (sheet 11). Sheet 12: List of enriched GO terms in DETs (based on the DETs listed in sheet 8–11). Sheet 13: Kegg pathways identified in DETs (based on the DETs listed in sheet 8). Sheet 14: List of different types of TRPs identified in the assembled transcriptome. Sheet 15: List of olfactory genes identified in the assembled transcriptome. Sheet 16: Genes/enzymes of carotenoid pigmentation pathway. Sheet 17: Genes/enzymes involved in juvenile and ecdysone pathway. Sheet 18: Gene involved in innate immune pathways. Sheet 19: Gene involved in sex-determining pathway. (XLSX 16726 kb)
Additional file 2:
**Figure S1.** Volcano plots showing the expression difference of transcripts between males and females, and between males and each female morph (based on log_2_ fold change- and p-values listed in Additional file 1: Sheet 4–7). **Figure S2.** Carotenoid pigmentation pathway. **Figure S3.** Juvenile hormone biosynthesis pathway. **Figure S4.** Sex determination pathway. (DOCX 265 kb)

## Abbreviations

CEGMA: Core eukaryotic gene mapping approach
CSPs: Chemosensory proteins
DAP: Meso-diaminopimelic acid
DEGs: Differentially expressed genes
DETs: Differentially expressed transcripts
DREDD: Death related ced-3/nedd2-like caspase
Dsx: Doublesex
FADD: Fas-associated death domain
FDR: False discovery rate
Fru: Fruitless
GNBPs: Gram-negative binding proteins
GO: Gene ontology
Hop: Hopscotch
Imd: Immune deficiency
IRs: Ionotropic receptors
JAK/STAT: Janus kinase and the signal transducer and activator of transcription
KEGG: Kyto encyclopaedia of genes and genomes
NCBI: National Center for Biotechnology Information
OBPs: Odorant binding proteins
ORs: Odorant receptors
PGRP-LC: Peptidoglycan recognition protein long class
PGRPs: Peptidoglycan recognition proteins
Sxl: Sex lethal
Tra: Transformer-2
UPPNEX: Uppsala Multidisciplinary Center for Advanced Computational Science
