# Supplementary material for: Transcriptome profiling in the damselfly Ischnura elegans identifies genes with sex-biased expression
Source: BMC Genomics. 2016 Dec 1;17:985. doi: 10.1186/s12864-016-3334-6 (PMC5131402; doi:10.1186/s12864-016-3334-6)

# Figure S1: Volcano plots showing the expression difference of transcripts between males and females, and between males and each female morph (based on log_2_ fold change- and p-values listed in Additional file 1, sheet 4-7).


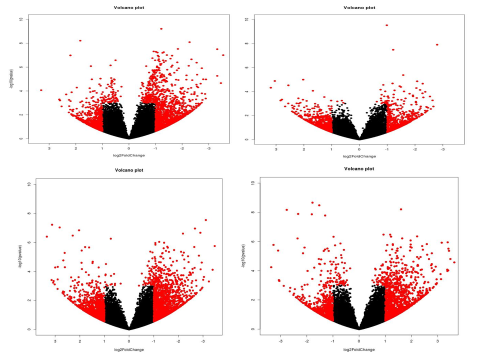


Figure S2 Carotenoid Pigmentation Pathway. Black colour denotes the substrate/product and red colour represents genes/enzymes.
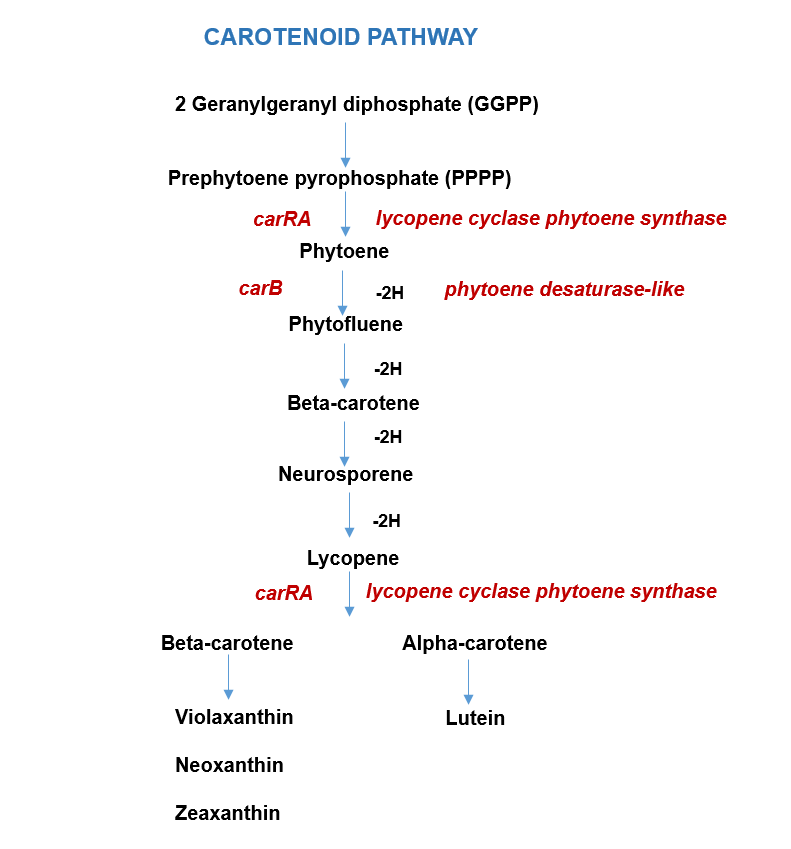


Note:

**bifunctional lycopene cyclase phytoene synthase-like**: up-regulated in males against androchrome

**lycopene cyclase phytoene synthase**: up-regulated in infuscans

Figure S3 Juvenile hormone biosynthesis pathway. Black colour denotes the substrate/product and red colour represents genes/enzymes.


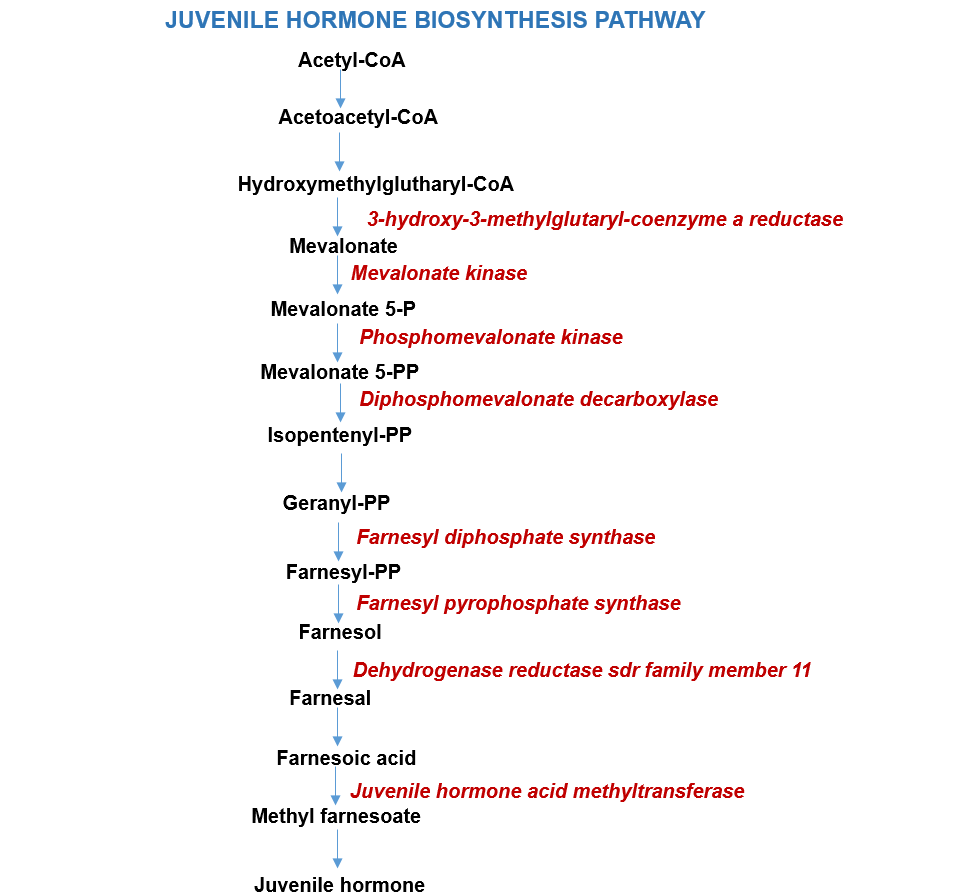


Note:

**juvenile hormone acid methyltransferase**: up-regulated in males against androchrome

Figure S4: Sex determination pathway. Black colour represents genes identified in assembled transcriptome.


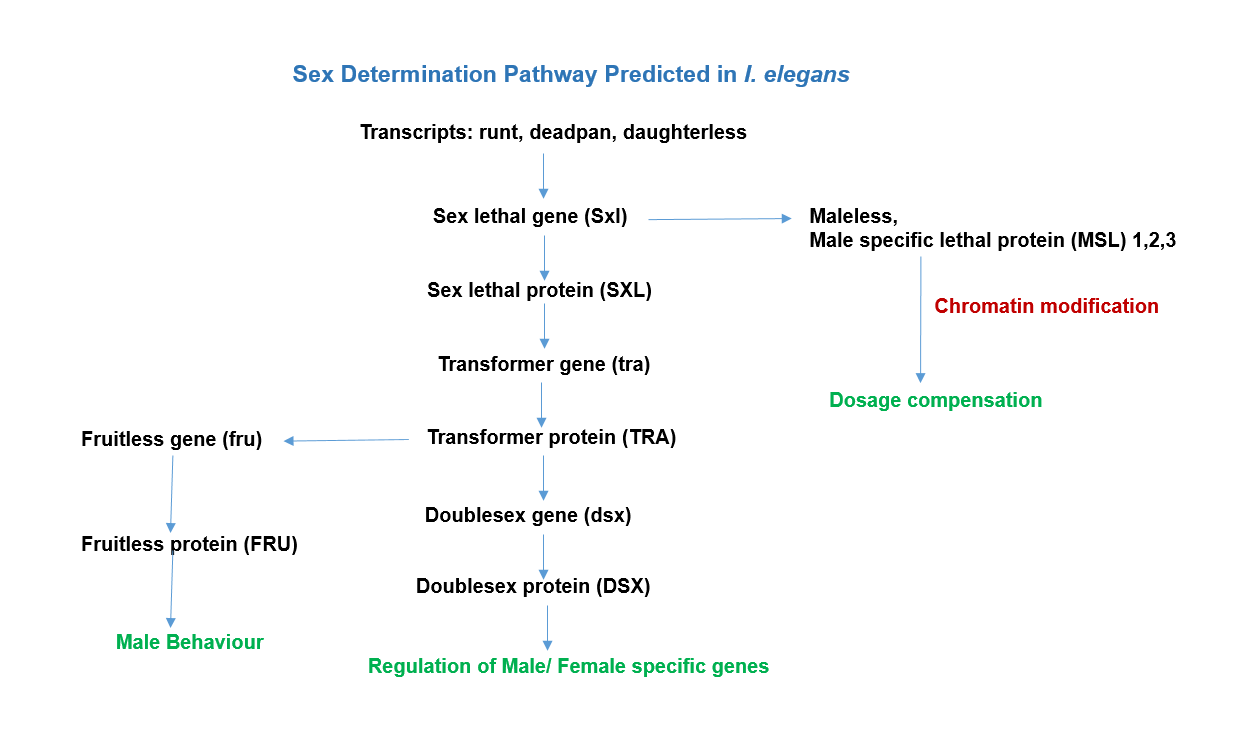

Supplement: Additional file 2: — Figure S1. Volcano plots showing the expression difference of transcripts between males and females, and between males and each female morph (based on log2 fold change- and p-values listed in Additional file 1: Sheet 4–7). Figure S2. Carotenoid pigmentation pathway. Figure S3. Juvenile hormone biosynthesis pathway. Figure S4. Sex determination pathway. (DOCX 265 kb) [file 12864_2016_3334_MOESM2_ESM.docx]
